# Effect of Supervised Versus Hybrid Delivery of Physiotherapeutic Scoliosis-Specific Exercises in Adolescents with Idiopathic Scoliosis: Systematic Review and Meta-Analysis

**DOI:** 10.3390/medicina62040768

**Published:** 2026-04-15

**Authors:** Su-Young Lee, Ju-Young Tak

**Affiliations:** 1 Department of Physical Therapy, Graduate School, Daegu University, Jillyang, Gyeongsan 712-714, Republic of Korea; 2 Department of Physical Therapy, Daegu Catholic University, Hayang, Gyeongsan 38453, Republic of Korea

**Keywords:** adolescent idiopathic scoliosis, physiotherapeutic scoliosis-specific exercises, therapist supervision, Cobb angle, angle of trunk rotation, SRS-22, meta-analysis, randomized controlled trials

## Abstract

*Background and Objectives*: Adolescent idiopathic scoliosis (AIS) is a three-dimensional spinal deformity that requires effective conservative management. Physiotherapeutic scoliosis-specific exercises (PSSE) have been widely used; however, evidence regarding their effectiveness according to therapist supervision intensity remains limited. Therefore, this study aimed to evaluate the effects of PSSE in patients with AIS and to examine differences according to supervision intensity. *Design*: Systematic review and meta-analysis of randomized controlled trials (RCTs) conducted in accordance with the PRISMA 2020 guidelines. *Materials and Methods*: RCTs involving patients with AIS (aged 10–18 years, Cobb angle 10–45°) were included if PSSE was applied alone or in combination with other conservative treatments. The primary outcomes were Cobb angle, angle of trunk rotation (ATR), and Scoliosis Research Society-22 (SRS-22). Effect sizes were calculated as standardized mean differences (SMDs) using a random-effects model. Subgroup analyses were performed according to supervision intensity. *Results*: A total of 10 RCTs (*n* = 600) were included. The pooled analysis demonstrated that PSSE significantly reduced Cobb angle (SMD = −0.52, 95% CI −0.79 to −0.25, *p* < 0.001) and ATR (SMD = −1.01, 95% CI −1.53 to −0.48, *p* < 0.001) compared with control interventions. In subgroup analyses, fully supervised interventions showed larger and more consistent effects, with statistically significant improvements in both Cobb angle (SMD = −0.70) and ATR (SMD = −1.33), whereas hybrid approaches did not demonstrate statistically significant effects. However, statistical support for subgroup differences was stronger for ATR than for Cobb angle. SRS-22 scores showed a trend toward improvement but did not reach statistical significance. Moderate to high heterogeneity was observed in some analyses, and risk-of-bias concerns were identified in several studies. *Conclusions*: PSSE may be an effective conservative intervention for improving structural curvature and trunk rotation in patients with AIS. Subgroup findings suggest that closer therapist supervision may be associated with more favorable effects, particularly for ATR; however, these findings should be interpreted cautiously because of heterogeneity and potential risk of bias. Large-scale, high-quality trials are warranted to confirm the magnitude and long-term sustainability of clinical effects.

## 1. Introduction

Scoliosis is a complex three-dimensional (3D) structural deformity of the spine that involves not only lateral curvature but also vertebral rotation [1]. It affects the alignment of the vertebral bodies as well as the morphology of the rib cage and trunk, and is considered a multifactorial condition with diverse clinical manifestations [2]. When no clear underlying disease or identifiable cause is present, the condition is classified as idiopathic scoliosis (IS) [2].

Adolescent idiopathic scoliosis (AIS) is the most common form of IS and has been reported to occur in 0.47% to 12% of the general population [3]. Its prevalence is higher in females, and this sex difference becomes more pronounced as curve magnitude increases [4,5]. The risk of progression also varies according to the initial Cobb angle and skeletal maturity. Curves of 20° or less have a relatively low risk of progression, whereas curves greater than 20° in skeletally immature patients are associated with a substantially higher risk [6]. These features highlight the importance of early detection and appropriate conservative management.

Treatment of AIS is broadly classified into conservative and non-conservative approaches, and non-surgical conservative management is generally recommended as the first-line treatment strategy for most patients [2]. Conservative treatment includes bracing and exercise therapy. The primary goal of AIS management is to prevent curve progression by maintaining the Cobb angle below 30°, and this objective can be categorized into morphological and functional goals [7]. Furthermore, treatment requires a multidimensional approach that includes preventing or reducing curve progression, maintaining and improving respiratory function, preventing pain, and enhancing aesthetic appearance [2]. The Cobb angle is the most widely used outcome measure for evaluating treatment effects, serving as the standard radiographic parameter that quantitatively reflects the extent of structural deformity [8]. In addition, the Angle of Trunk Rotation (ATR) is measured using a scoliometer during the forward bending test and is utilized as a clinical parameter to indirectly assess external trunk deformity [9]. More recently, the Scoliosis Research Society-22 (SRS-22) questionnaire, a disease-specific instrument for assessing health-related quality of life in patients with idiopathic scoliosis, has been widely used as an important outcome measure [10].

Physiotherapeutic scoliosis-specific exercises (PSSE) are asymmetric, individualized exercise interventions designed according to each patient’s specific curve pattern and three-dimensional deformity characteristics [7]. PSSE incorporates active postural awareness, self-correction, breathing control, and the integration of corrected posture into activities of daily living as its core components [2]. The SOSORT (International Society on Scoliosis Orthopaedic and Rehabilitation Treatment) recommends PSSE as a key conservative treatment strategy for patients with AIS [2], PSSE encompasses various approaches, including the Schroth method, SEAS (Scientific Exercise Approach to Scoliosis), BSPTS (Barcelona Scoliosis Physical Therapy School), Lyon method, and FITS method. These approaches share common principles that emphasize three-dimensional self-correction, trunk stabilization, respiratory training, and functional postural re-education [7].

According to previous studies, PSSE has demonstrated beneficial effects on Cobb angle progression, trunk asymmetry, and certain patient-reported outcomes in adolescents with idiopathic scoliosis (AIS) [11,12,13]. However, previous systematic reviews and extended literature reviews have primarily focused on the overall effectiveness of PSSE and have not clearly distinguished how differences in study design and intervention delivery may influence treatment outcomes. In particular, earlier syntheses often included heterogeneous study designs, did not focus exclusively on randomized controlled trials (RCTs), and did not specifically examine therapist supervision intensity as a potential effect modifier. In addition, recently published eligible trials have not always been incorporated into prior evidence syntheses. Therefore, an updated review focusing specifically on RCTs and supervision intensity is warranted.

Therefore, the aim of this study was to systematically evaluate the effects of PSSE on spinal deformity and quality of life in patients with AIS by synthesizing evidence from randomized controlled trials only. In particular, this review sought to clarify whether differences in supervision intensity may influence treatment effectiveness and thereby provide clinically relevant evidence for optimizing PSSE delivery.

## 2. Methods

This study followed the Preferred Reporting Items for Systematic Reviews and Meta-Analyses (PRISMA) 2020 statement [14]. The review protocol was registered in PROSPERO, the International Prospective Register of Systematic Reviews (registration number: CRD420261288470). Because registration was completed after the review had commenced, it should be considered retrospective. No major deviations from the registered protocol affected the eligibility criteria, primary outcomes, or overall analytical approach.

### 2.1. Eligibility Criteria

The inclusion criteria were as follows: (1) randomized controlled trials (RCTs); (2) studies involving adolescents aged 10–18 years at baseline with a clinical and radiographic diagnosis of adolescent idiopathic scoliosis (AIS), and a baseline Cobb angle ranging from 10° to 45°; (3) studies in which physiotherapeutic scoliosis-specific exercises (PSSE) were applied either as a standalone intervention or in combination with other conservative treatments (e.g., bracing), including approaches such as the Schroth method, SEAS, and BSPTS; (4) studies in which the control group received usual care, bracing, traditional exercise, or other non–scoliosis-specific exercise programs; (5) studies reporting at least one of the following primary outcome measures: Cobb angle, angle of trunk rotation (ATR), or the Scoliosis Research Society-22 (SRS-22) questionnaire; (6) studies providing sufficient statistical data to enable the calculation of effect sizes; (7) studies published in English. For borderline cases, eligibility was determined based on the study’s baseline participant characteristics and population definition rather than the reported mean age alone. The upper age limit of 18 years was chosen to reflect the conventional definition of adolescence and to include eligible trials in which participants were classified as adolescents at baseline.

The exclusion criteria were as follows: (1) studies involving non-idiopathic scoliosis (e.g., congenital or neuromuscular scoliosis); (2) studies including surgical interventions; (3) non-experimental study designs, such as single-group trials without a control group, observational studies, case reports, qualitative studies, or animal experiments; (4) gray literature not formally published in peer-reviewed journals (e.g., conference abstracts or posters); (5) duplicate publications; (6) studies that did not provide sufficient data for effect size calculation; (7) studies containing major errors in tables or figures that precluded accurate interpretation of the results.

### 2.2. Search Strategy and Study Selection

Four electronic databases—PubMed, Embase, the Cochrane Library, and Web of Science—were systematically searched from their inception to 14 January 2026. The entire process of literature search and study selection was conducted systematically in accordance with the Preferred Reporting Items for Systematic Reviews and Meta-Analyses (PRISMA) 2020 statement and was structured around the PRISMA flow diagram [14]. The search strategy was specifically designed to focus on adolescent idiopathic scoliosis (AIS) and physiotherapeutic scoliosis-specific exercises (PSSE).

**Figure 1:**
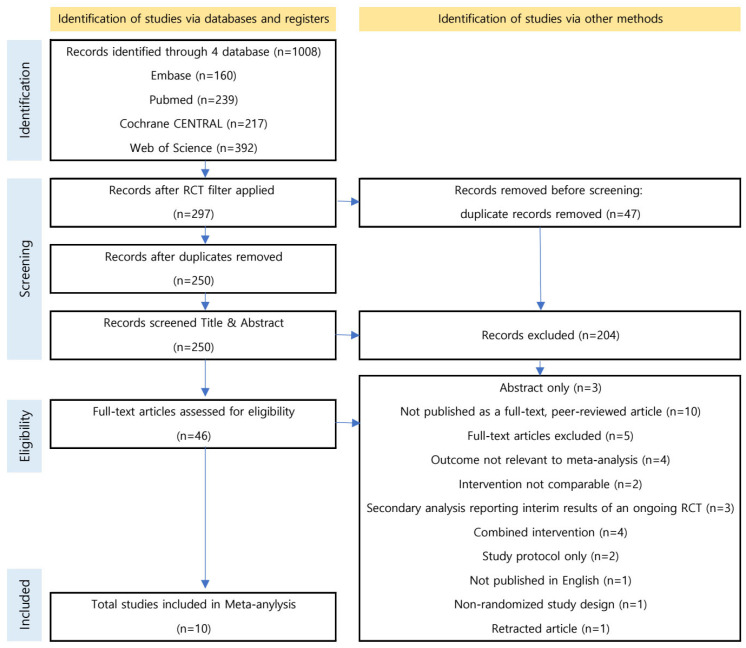
Flowchart of study selection.

A total of 1008 records were identified through database searching (Figure 1). After application of the RCT filter and removal of 47 duplicate records, 250 records remained for title and abstract screening. Of these, 204 were excluded. The remaining 46 full-text articles were assessed for eligibility, and 36 were excluded after full-text review. Ultimately, 10 studies were included in the systematic review and meta-analysis.

The search terms were structured into three conceptual blocks. The first block included disease-related terms (“scoliosis,” “adolescent idiopathic scoliosis,” and “AIS”); the second block comprised intervention-related terms (“Schroth,” “SEAS,” “BSPTS,” “PSSE,” “scoliosis-specific exercise,” and “physiotherapeutic scoliosis-specific exercise”); and the third block included age-related terms (“adolescent,” “teen,” and “youth”). The search strategy was tailored to the characteristics of each database by combining controlled vocabulary (e.g., MeSH and Emtree terms) with free-text keywords.

The search results were exported to a reference management software program, and duplicate records were removed. Subsequently, the titles and abstracts were screened independently by two reviewers, and full-text articles were independently assessed for eligibility. Any disagreements during the selection process were resolved through discussion and consensus. In addition, the reference lists of the included studies and relevant articles were manually reviewed to minimize the risk of missing potentially eligible studies. Only studies published in English were included, and no additional grey literature search was conducted. These restrictions may have limited the comprehensiveness of the review and should be considered when interpreting the findings.

### 2.3. Study Selection and Data Extraction

Study selection was conducted based on predefined inclusion and exclusion criteria. Retrieved records were initially screened by reviewing titles and abstracts, followed by full-text assessment of studies considered potentially eligible to determine final inclusion. Title and abstract screening and full-text eligibility assessment were performed independently by two reviewers. Any disagreements were resolved through discussion and consensus.

Data extraction was conducted primarily by one reviewer using a standardized extraction form based on predefined criteria. When uncertainty, inconsistency, or the need for verification arose during extraction, the extracted information was reviewed and discussed with the co-investigator until consensus was reached. However, data extraction was not performed independently in duplicate, which may have increased the risk of selection bias and information bias related to the extraction process. The extracted variables included the first author and year of publication; country of study; study design; total number of participants and sex distribution; mean age; baseline Cobb angle; scoliosis type and major curve location; type and detailed description of the intervention; duration of each exercise session; total intervention period; weekly session frequency; supervision modality (e.g., fully supervised or hybrid/home-based); details of the control intervention; outcome measures (Cobb angle, angle of trunk rotation [ATR], and Scoliosis Research Society-22 [SRS-22]); follow-up duration; and reports of adverse events or side effects. For systematic management of the literature and data organization, EndNote 21 and Microsoft Excel 2021 were utilized.

In this study, physiotherapeutic scoliosis-specific exercises (PSSE) were defined as asymmetric, individualized exercise interventions designed according to each patient’s specific curve pattern and three-dimensional deformity characteristics [15]. PSSE was characterized as an intervention incorporating active self-correction, three-dimensional postural control, breathing regulation, trunk stabilization, and functional postural re-education [2,11]. Exercise approaches aimed at structural correction—such as the Schroth method, SEAS, and BSPTS—were included [2], whereas general calisthenics or symmetrical strengthening exercises were not considered PSSE.

In addition, to evaluate differences in effectiveness according to the level of supervision of PSSE interventions, the included studies were categorized based on supervision intensity. For the subgroup analyses, supervision intensity was defined a priori according to the proportion, frequency, and continuity of therapist-supervised contact throughout the intervention period, rather than according to delivery mode alone. Interventions were classified as fully supervised when all or most exercise sessions were directly supervised by a therapist on a regular basis throughout the intervention period, typically with repeated correction and feedback to support exercise fidelity. Interventions were classified as hybrid/home-based when supervised instruction was combined with substantial home-based, remote, or digitally delivered exercise, or when direct therapist supervision was limited to only part of the intervention period. Thus, supervision intensity in this review was intended to capture not only the amount of therapist contact, but also the degree of real-time correction, adherence monitoring, and fidelity support provided during PSSE delivery. Session frequency and duration were extracted as descriptive indicators of intervention intensity, but classification was not based on session frequency alone. Because reporting of supervision structure was not fully standardized across the included trials, these categories should be understood as pragmatic clinical groupings rather than strict dose-based thresholds. Subgroup analyses were performed according to this prespecified classification.

### 2.4. Outcome Measures

The primary outcome measure of this study was the Cobb angle, reflecting the structural deformity of the spine. Secondary outcome measures included the angle of trunk rotation (ATR), which assesses trunk rotational deformity, and the Scoliosis Research Society-22 (SRS-22) questionnaire, which evaluates health-related quality of life.

Separate meta-analyses were conducted for each outcome (Cobb angle, ATR, and SRS-22). In addition, subgroup analyses were performed to examine differences in effectiveness according to the level of supervision of PSSE interventions by categorizing studies into fully supervised and hybrid/home-based interventions.

### 2.5. Assessment of Risk of Bias and Quality of Evidence

The methodological quality of the included randomized controlled trials was assessed using the Cochrane Risk of Bias 2 (RoB 2) tool [16]. Risk-of-bias assessment was also conducted primarily by one reviewer and was not independently performed in duplicate. Although uncertain judgments were reviewed with the co-investigator, the absence of fully independent duplicate assessment may have introduced subjectivity and increased the risk of information bias in methodological quality judgments. This should be considered a methodological limitation when interpreting the risk-of-bias assessments. Each study was evaluated across the following five domains: (1) bias arising from the randomization process; (2) bias due to deviations from intended interventions; (3) bias due to missing outcome data; (4) bias in the measurement of outcomes; and (5) bias in the selection of the reported results. When judgment was uncertain, the assessment was discussed with a co-investigator to reach a final decision. Each domain was classified as “low risk,” “some concerns,” or “high risk,” and an overall risk of bias was determined accordingly. In accordance with the RoB 2 guidance, studies were judged as having an overall high risk of bias if at least one domain was rated as high risk. If no domain was rated as high risk but at least one domain raised some concerns, the study was classified as having an overall rating of “some concerns” [16].

The certainty of evidence was assessed using the Grading of Recommendations Assessment, Development and Evaluation (GRADE) approach [17], and the level of evidence was classified into four categories: high, moderate, low, and very low [18]. The level of evidence was downgraded based on five domains: risk of bias, inconsistency, indirectness, imprecision, and publication bias [18]. The certainty of evidence was assessed independently for each primary outcome (Cobb angle, ATR, and SRS-22). A Summary-of-Findings table was prepared as Supplementary Table S1 to present the certainty of evidence for the main outcomes and the rationale for downgrading across the GRADE domains. In addition, the main text briefly summarizes the key domains contributing to downgrading for each outcome to improve transparency.

### 2.6. Statistical Analysis

Meta-analyses were performed using Review Manager (RevMan) version 5.4 (Cochrane Collaboration) [19]. A random-effects model was applied for all outcomes to account for potential clinical and methodological heterogeneity among studies [19]. Effect sizes were calculated as standardized mean differences (SMDs) with 95% confidence intervals (CIs) using the inverse-variance method, and Hedges’ g was applied to adjust for small-sample bias. Only studies providing sufficient numerical data for effect size calculation were included in the meta-analysis. Reported summary statistics were used as presented in the original articles, and no additional imputation or derivation of missing standard deviations was performed.

When a study reported separate outcomes for different curve regions (e.g., thoracic and lumbar Cobb angles), these were extracted separately as distinct outcome data when relevant to the predefined review outcomes. Therefore, the number of effect size estimates could exceed the number of included studies. These estimates reflected separate anatomical outcome data rather than multiple intervention-versus-shared-control comparisons within the same pooled analysis; accordingly, no splitting of the control group or combining of intervention arms was required. However, because curve-specific estimates derived from the same study may not be fully independent, these findings should be interpreted with caution. To examine the potential impact of this issue, sensitivity analyses were conducted for Cobb angle and ATR using one estimate per study when multiple estimates were reported. In these analyses, the major or primary curve was prioritized when explicitly identified in the original study; if not specified, the estimate corresponding to the curve with the larger baseline Cobb angle was selected.

The magnitude of effect sizes was interpreted according to Cohen’s criteria, with values <0.2 considered small, 0.2–0.5 considered moderate, and ≥0.5 considered large effects. Statistical significance was set at *p* < 0.05 [20].

Statistical heterogeneity among studies was assessed using Cochran’s Q test and the I^2^ statistic [19]. I^2^ values were interpreted as follows: <25% indicating low heterogeneity, 25–50% moderate heterogeneity, 50–75% substantial heterogeneity, and >75% considerable heterogeneity. When substantial or considerable heterogeneity was observed, subgroup analyses were conducted to explore potential sources of heterogeneity.

In this study, subgroup analyses were conducted to evaluate differences in effectiveness according to the level of supervision of PSSE interventions. Studies were categorized a priori as fully supervised or hybrid/home-based according to the proportion, frequency, and continuity of therapist-supervised contact throughout the intervention period, rather than delivery mode alone.

## 3. Results

### 3.1. Study Selection

A total of 10 randomized controlled trials (RCTs) that met the predefined eligibility criteria were ultimately included in this systematic review and meta-analysis (Figure 1). A total of 600 adolescents with idiopathic scoliosis were included in the analysis. The general characteristics of the included studies and details of the intervention protocols are summarized in Table 1.

### 3.2. Characteristics of Study and Participant

A total of 10 randomized controlled trials (RCTs), comprising 600 participants, were included in this meta-analysis [12,13,21,22,23,24,25,26,27,28]. The included studies were published between 2016 and 2025. The countries in which the studies were conducted were diverse: Turkey (*n* = 4), Serbia (*n* = 1), Iran (*n* = 1), Greece (*n* = 1), Egypt (*n* = 1), Canada (*n* = 1), and China (*n* = 1). The sample sizes of the individual studies ranged from 30 to 128 participants.

All included studies implemented physiotherapeutic scoliosis-specific exercises (PSSE) as the intervention. The types of interventions varied and included Schroth exercises alone (*n* = 4), Schroth combined with traditional exercise (*n* = 1), Schroth combined with bracing (*n* = 1), Schroth combined with spinal stabilization exercises (*n* = 1), SEAS-based intervention (*n* = 1), digitally delivered PSSE (*n* = 1), and other modified PSSE approaches (*n* = 1). The control groups received usual care, bracing, proprioceptive neuromuscular facilitation (PNF), core stabilization exercises, traditional exercise programs, or home-based exercise.

Based on supervision intensity, seven of the ten studies were classified as fully supervised, in which all sessions were directly supervised by a therapist. These studies delivered the intervention three times per week over a period ranging from 8 to 24 weeks, with individual session durations varying between 30 and 90 min. The remaining three studies were categorized as hybrid or partially supervised. These typically involved initial face-to-face supervision followed by home-based exercise, or a structure combining weekly supervised sessions with daily home exercise. In the digitally delivered PSSE study, participants received an initial supervised session and were subsequently instructed to perform home-based exercises at least five times per week.

The duration of the interventions varied across studies: 8 weeks (*n* = 2), 10–12 weeks (*n* = 3), 16 weeks (*n* = 1), and 24 weeks (*n* = 4). Most studies implemented three supervised sessions per week. In the fully supervised studies, the frequency and duration of supervision were relatively consistent. In contrast, the hybrid studies showed variability in supervision intensity and the proportion of home-based exercise.

All included studies assessed the Cobb angle as the primary outcome measure, while ATR and SRS-22 were the most frequently reported secondary outcomes. Some studies additionally evaluated the Walter Reed Visual Assessment Scale (WRVAS), pulmonary function parameters (e.g., FVC and FEV1), trunk symmetry indices, as well as muscle strength and functional measures. Baseline Cobb angles were generally reported within a range of 15° to 35°, indicating that most studies involved patients with mild to moderate AIS. Baseline mean ages were also generally consistent with an adolescent AIS population. In cases where the reported mean age approached the upper limit, eligibility was determined based on the study population definition and baseline inclusion criteria rather than the reported mean age alone. Likewise, the Cobb angle criterion was applied at baseline study entry, although reported study-level means and distributions varied modestly across trials.

Some key baseline variables were not consistently reported across the included studies and were therefore labeled as “not reported” in Table 1. In particular, information on curve location, skeletal maturity, and certain clinical descriptors was missing in several studies, which limited more detailed comparison of baseline characteristics and may have contributed to residual clinical heterogeneity.

Overall, the included studies differed in intervention type and supervision intensity, which provided the primary rationale for conducting subgroup analyses based on supervision modality.

### 3.3. Risk of Bias in Studies

Overall, three studies (Büyükturan 2024; Kaya 2025; Mohamed 2021) [21,23,26] were rated as low risk across all domains and were therefore classified as having an overall low risk of bias. Five studies (Khaledi 2024; Kocaman 2021; Schreiber 2016; Yagci 2019; Yuan 2025) [12,13,24,27,28] were judged as having some concerns in at least one domain but no high-risk ratings, and were consequently classified as overall “some concerns.” The remaining two studies (Dimitrijevic 2025; Kyrkousis 2024) [22,25] were rated as high risk in the domain of bias due to deviations from intended interventions (Domain 2), resulting in an overall high risk of bias classification.

**Figure 2:**
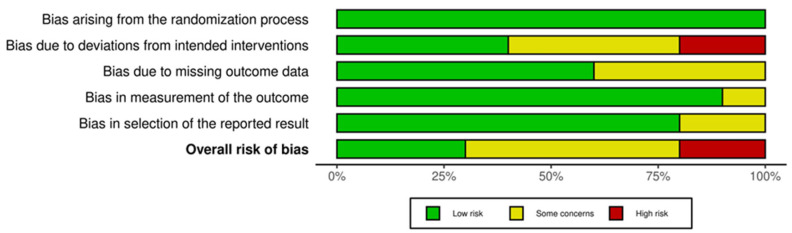
Risk-of-bias summary of the included studies based on the Cochrane RoB 2 tool.

**Figure 3:**
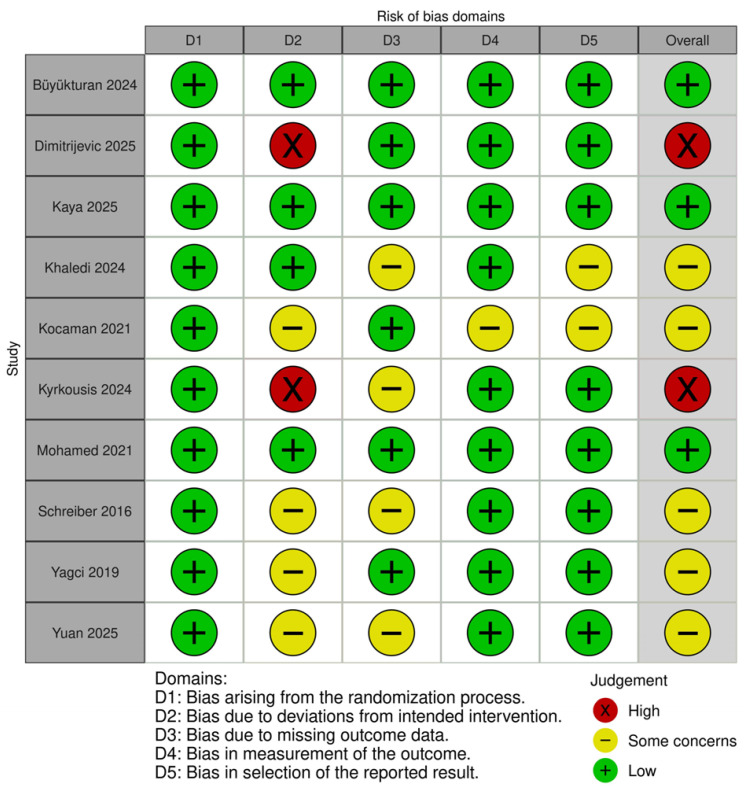
Overall risk-of-bias judgments of the included studies based on the Cochrane RoB 2 tool [12,13,21,22,23,24,25,26,27,28].

In summary, approximately 30% of the included studies were rated as low risk, 50% as some concerns, and 20% as high risk (Figure 2 and Figure 3).

1. Bias arising from the randomization process (Domain 1) was rated as low risk in all included studies.
2. Bias due to deviations from intended interventions (Domain 2) demonstrated the greatest variability across studies. Seven studies were rated as low risk, one study as having some concerns, and two studies as high risk. This was likely related to the inherent difficulty of blinding participants and therapists in exercise-based intervention studies.
3. Bias due to missing outcome data (Domain 3) was rated as low risk in seven studies, while three studies were judged as having some concerns. In several cases, reporting regarding loss to follow-up or the handling of missing data was insufficient.
4. Bias in the measurement of outcomes (Domain 4) was rated as low risk in nine studies and as having some concerns in one study. This finding may be attributed to the use of objective radiographic measures, such as the Cobb angle.
5. Bias in the selection of the reported results (Domain 5) was rated as low risk in seven studies and as having some concerns in three studies, with no study classified as high risk in this domain.

### 3.4. Overall Effects of PSSE Compared with Control

#### 3.4.1. Overall Effect on Cobb Angle

**Figure 4:**
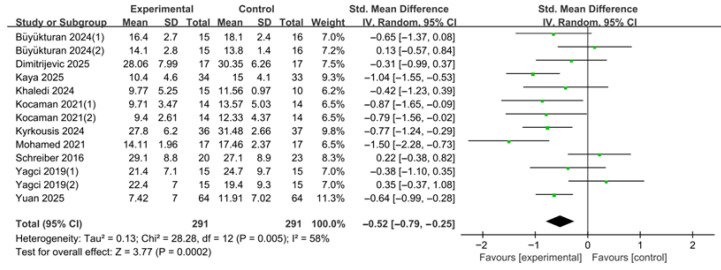
Effects of PSSE on Cobb angle. Forest plot showing standardized mean differences (SMDs) with 95% confidence intervals based on a random-effects model. Negative SMD values favor PSSE [12,13,21,22,23,24,25,26,27,28]. The green dots represent the effect estimates of individual studies, and the black diamond represents the pooled overall effect. (1) and (2) indicate separate curve-specific estimates extracted from the same study.

Based on 13 effect size estimates derived from 10 studies, the overall effect of various forms of PSSE on the Cobb angle was analyzed (Figure 4). In some analyses, the number of effect size estimates exceeded the number of included studies because separate curve-specific outcomes reported within a study were extracted individually. Standardized mean differences (SMDs) were calculated to estimate effect sizes. A random-effects model was applied to account for potential clinical and methodological heterogeneity across studies.

The pooled analysis demonstrated that PSSE significantly reduced the Cobb angle compared with the control group (SMD = −0.52, 95% CI −0.79 to −0.25). The overall effect was statistically significant (Z = 3.77, *p* = 0.0002). Between-study heterogeneity was moderate (τ^2^ = 0.13), and Cochran’s Q test indicated statistically significant heterogeneity (χ^2^ = 28.28, df = 12, *p* = 0.005). The I^2^ value was 58%, suggesting substantial variability in effect sizes among the included studies.

To assess the potential impact of including multiple curve-specific estimates from the same study, a sensitivity analysis was conducted using one estimate per study when multiple estimates were reported. In this sensitivity analysis, the pooled effect remained statistically significant in favor of the experimental group (SMD = −0.63, 95% CI −0.90 to −0.36; Z = 4.63, *p* < 0.00001). Between-study heterogeneity was reduced to a moderate level (τ^2^ = 0.09; χ^2^ = 17.37, df = 9, *p* = 0.04; I^2^ = 48%). These findings suggest that the overall Cobb angle result was not materially affected by the handling of multiple estimates derived from the same study.

An additional sensitivity analysis excluding studies judged as overall high risk of bias was also conducted. The pooled effect remained statistically significant in favor of the PSSE group (SMD = −0.51, 95% CI −0.83 to −0.19; Z = 3.09, *p* = 0.002), and the magnitude and direction of effect were essentially unchanged compared with the main analysis. Heterogeneity remained substantial (τ^2^ = 0.18; χ^2^ = 26.97, df = 10, *p* = 0.003; I^2^ = 63%). These findings suggest that the overall effect on Cobb angle was robust to the exclusion of high-risk studies.

#### 3.4.2. Overall Effect on ATR

**Figure 5:**
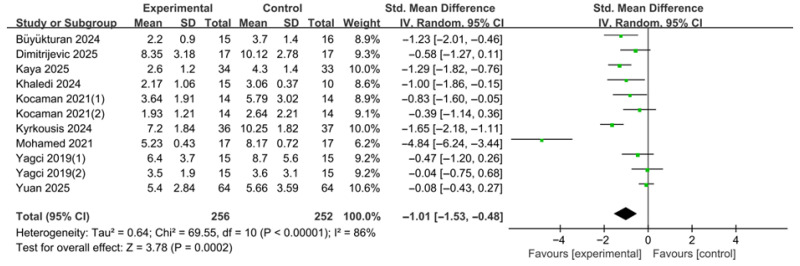
Effects of PSSE on angle of trunk rotation (ATR). Forest plot showing standardized mean differences (SMDs) with 95% confidence intervals based on a random-effects model. Negative SMD values favor PSSE [12,21,22,23,24,25,26,27,28]. The green dots represent the effect estimates of individual studies, and the black diamond represents the pooled overall effect. (1) and (2) indicate separate curve-specific estimates extracted from the same study.

Based on 11 effect size estimates derived from nine studies, the overall effect of various forms of PSSE on the angle of trunk rotation (ATR) was analyzed (Figure 5). The number of effect size estimates exceeded the number of included studies because some studies reported separate anatomical outcome data that were extracted individually. Standardized mean differences (SMDs) were calculated to estimate effect sizes. A random-effects model was applied to account for potential clinical and methodological heterogeneity across studies.

The pooled analysis showed that PSSE significantly reduced the angle of trunk rotation (ATR) compared with the control group (SMD = −1.01, 95% CI −1.53 to −0.48). The test for overall effect indicated a statistically significant difference (Z = 3.78, *p* = 0.0002). Between-study heterogeneity was high (τ^2^ = 0.64), and Cochran’s Q test revealed statistically significant heterogeneity (χ^2^ = 69.55, df = 10, *p* < 0.00001). The I^2^ value was 86%, indicating considerable variability in effect sizes among the included studies.

The substantial heterogeneity observed in this analysis may reflect clinical and methodological differences across studies, including variations in concurrent brace use, baseline curve severity, intervention duration, PSSE approach, and supervision modality.

To assess the potential impact of including multiple curve-specific estimates from the same study, a sensitivity analysis was conducted using one estimate per study when multiple estimates were reported. The pooled effect remained statistically significant in favor of the experimental group (SMD = −1.20, 95% CI −1.81 to −0.59; Z = 3.84, *p* = 0.0001), although heterogeneity remained considerable (τ^2^ = 0.73; χ^2^ = 64.24, df = 8, *p* < 0.00001; I^2^ = 88%). These findings suggest that the overall ATR result was not materially affected by the handling of multiple estimates derived from the same study.

An additional sensitivity analysis excluding studies judged as overall high risk of bias was also conducted. The pooled effect remained statistically significant in favor of the PSSE group (SMD = −0.99, 95% CI −1.59 to −0.38; Z = 3.20, *p* = 0.001), and the magnitude and direction of effect were essentially unchanged compared with the main analysis. Heterogeneity remained considerable (τ^2^ = 0.70; χ^2^ = 57.09, df = 8, *p* < 0.00001; I^2^ = 86%). These findings suggest that the overall ATR effect was robust to the exclusion of high-risk studies, although substantial heterogeneity persisted.

#### 3.4.3. Overall Effect on SRS-22

**Figure 6:**
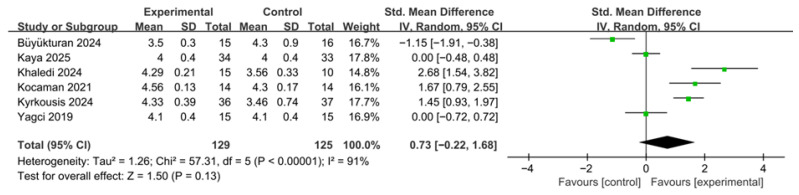
Effects of PSSE on Scoliosis Research Society-22 (SRS-22) scores. Forest plot showing standardized mean differences (SMDs) with 95% confidence intervals based on a random-effects model. Positive SMD values favor PSSE [12,21,23,24,25,27]. The green dots represent the effect estimates of individual studies, and the black diamond represents the pooled overall effect.

A total of six studies were included in the meta-analysis of the overall Scoliosis Research Society-22 (SRS-22) score. The included studies compared changes in quality of life between the PSSE intervention groups and control groups (Figure 6). To account for differences in scoring ranges and variability across studies, effect sizes were calculated as standardized mean differences (SMDs). A random-effects model was applied to reflect potential clinical and methodological heterogeneity among the included studies.

The meta-analysis showed that PSSE tended to improve SRS-22 scores compared with the control group; however, the difference did not reach statistical significance (SMD = 0.73, 95% CI −0.22 to 1.68). The test for overall effect was not statistically significant (Z = 1.50, *p* = 0.13). Between-study heterogeneity was very high (τ^2^ = 1.26), and Cochran’s Q test indicated statistically significant heterogeneity (χ^2^ = 57.31, df = 5, *p* < 0.00001). The I^2^ value was 91%, demonstrating considerable variability in effect sizes among the included studies.

The very high heterogeneity observed for this outcome may reflect differences in intervention duration, PSSE type, concurrent treatments such as bracing, supervision modality, and the relatively small number of studies available for pooling.

An additional sensitivity analysis excluding studies judged as overall high risk of bias was conducted for the SRS-22 outcome. The pooled effect remained in favor of the PSSE group but did not reach statistical significance (SMD = 0.58, 95% CI −0.50 to 1.66). Compared with the main analysis, the direction of effect was unchanged, although the magnitude of effect was slightly attenuated. These findings suggest that the non-significant result for SRS-22 was not materially altered by the exclusion of high-risk studies. A summary of the sensitivity analyses for the main outcomes is provided in Supplementary Table S2.

### 3.5. Secondary Analysis: Effects According to Supervision Intensity

#### 3.5.1. Effects on Cobb Angle According to Supervision Intensity

**Figure 7:**
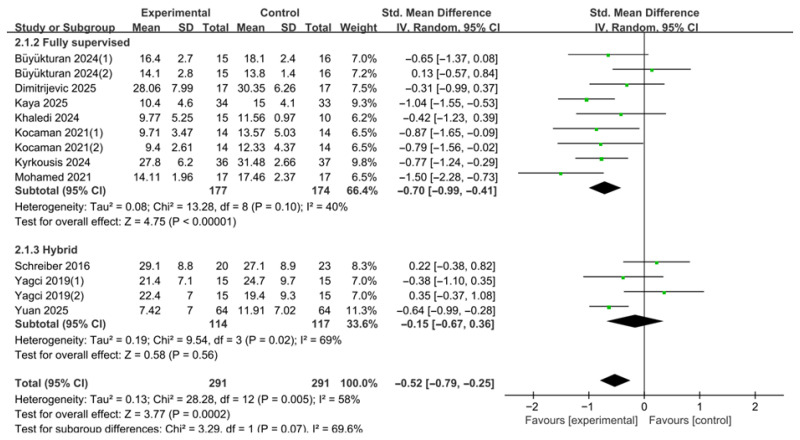
Subgroup analysis of Cobb angle according to supervision intensity. Forest plot showing standardized mean differences (SMDs) with 95% confidence intervals based on a random-effects model. Negative SMD values favor PSSE [12,13,21,22,23,24,25,26,27,28]. The green dots represent the effect estimates of individual studies, and the black diamond represents the pooled overall effect. (1) and (2) indicate separate curve-specific estimates extracted from the same study.

In the subgroup analysis, studies were categorized into fully supervised and hybrid/home-based groups according to the prespecified supervision classification based on the proportion, frequency, and continuity of therapist-supervised contact throughout the intervention period, rather than delivery mode alone (Figure 7). A total of 10 studies were included in this analysis, and a random-effects model was applied to account for between-study heterogeneity. In some analyses, the number of effect size estimates exceeded the number of included studies because separate curve-specific outcomes reported within a study were extracted individually.

The fully supervised subgroup comprised nine comparisons. The pooled results demonstrated that fully supervised PSSE interventions significantly reduced the Cobb angle compared with the control group (SMD = −0.70, 95% CI −0.99 to −0.41). The test for overall effect was statistically significant (Z = 4.75, *p* < 0.00001). Between-study heterogeneity was moderate (τ^2^ = 0.08; χ^2^ = 13.28, df = 8, *p* = 0.10; I^2^ = 40%).

The hybrid/home-based subgroup comprised four comparisons. The pooled results showed a reduction in Cobb angle; however, the effect was not statistically significant (SMD = −0.15, 95% CI −0.67 to 0.36). The test for overall effect was also not significant (Z = 0.58, *p* = 0.56). Between-study heterogeneity was high (τ^2^ = 0.19; χ^2^ = 9.54, df = 3, *p* = 0.02; I^2^ = 69%).

The test for subgroup differences indicated that the difference in effect sizes between the two supervision groups was not statistically significant (χ^2^ = 3.29, df = 1, *p* = 0.07). Therefore, although the fully supervised subgroup showed a larger pooled effect, this finding should be interpreted as suggestive rather than confirmatory, because the subgroup difference did not reach statistical significance.

#### 3.5.2. Effects on ATR According to Supervision Intensity

**Figure 8:**
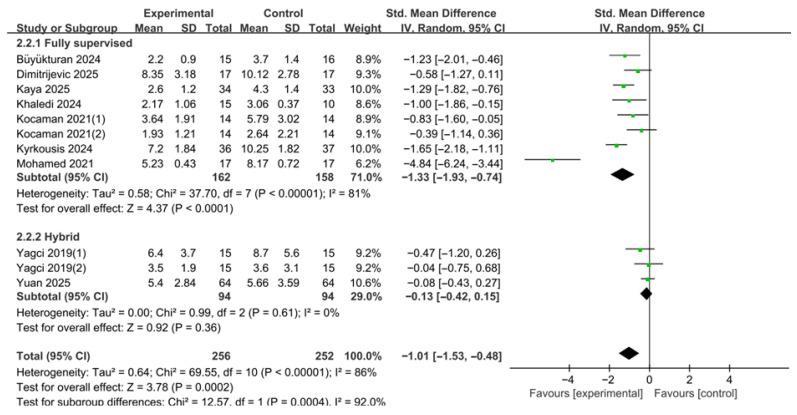
Subgroup analysis of angle of trunk rotation (ATR) according to supervision intensity. Forest plot showing standardized mean differences (SMDs) with 95% confidence intervals based on a random-effects model. Negative SMD values favor PSSE [12,13,21,22,23,24,25,26,27,28]. The green dots represent the effect estimates of individual studies, and the black diamond represents the pooled overall effect. (1) and (2) indicate separate curve-specific estimates extracted from the same study.

In the subgroup analysis of ATR according to supervision intensity, studies were categorized into fully supervised and hybrid/home-based groups according to the prespecified supervision classification based on the proportion, frequency, and continuity of therapist-supervised contact throughout the intervention period, rather than delivery mode alone (Figure 8). A total of 11 effect size estimates derived from nine studies were included in this analysis. A random-effects model was applied to account for between-study heterogeneity. This was because some studies provided separate curve-specific outcome data that were extracted as distinct effect size estimates.

The fully supervised subgroup comprised eight effect size estimates. The pooled results demonstrated that fully supervised PSSE interventions significantly reduced ATR compared with the control group (SMD = −1.33, 95% CI −1.93 to −0.74). The test for overall effect was statistically significant (Z = 4.37, *p* < 0.0001). Between-study heterogeneity was high (τ^2^ = 0.58; χ^2^ = 37.70, df = 7, *p* < 0.00001; I^2^ = 81%).

The hybrid/home-based subgroup comprised three effect size estimates. The pooled results showed a reduction in ATR; however, the effect was not statistically significant (SMD = −0.13, 95% CI −0.42 to 0.15). The test for overall effect was also not significant (Z = 0.92, *p* = 0.36). No significant heterogeneity was observed among these studies (τ^2^ = 0.00; χ^2^ = 0.99, df = 2, *p* = 0.61; I^2^ = 0%).

The test for subgroup differences indicated that the reduction in ATR differed significantly between the two supervision groups (χ^2^ = 12.57, df = 1, *p* = 0.0004). This finding provides stronger evidence that supervision intensity may influence ATR outcomes, although the result should still be interpreted cautiously in light of the substantial heterogeneity within the fully supervised subgroup.

#### 3.5.3. Effects on SRS-22 According to Supervision Intensity

**Figure 9:**
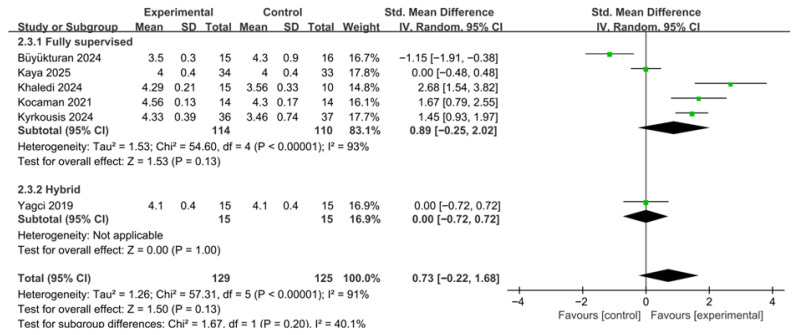
Subgroup analysis of Scoliosis Research Society-22 (SRS-22) scores according to supervision intensity. Forest plot showing standardized mean differences (SMDs) with 95% confidence intervals based on a random-effects model. Positive SMD values favor PSSE [12,21,23,24,25,27]. The green dots represent the effect estimates of individual studies, and the black diamond represents the pooled overall effect.

In the subgroup analysis of SRS-22 scores according to supervision intensity, studies were categorized into fully supervised and hybrid/home-based groups according to the prespecified supervision classification based on the proportion, frequency, and continuity of therapist-supervised contact throughout the intervention period, rather than delivery mode alone (Figure 9). A total of six studies were included in this analysis. A random-effects model was applied to account for between-study heterogeneity.

The fully supervised subgroup included five studies. The pooled results indicated that SRS-22 scores tended to improve in the fully supervised group compared with the control group; however, the difference was not statistically significant (SMD = 0.89, 95% CI −0.25 to 2.02). The test for overall effect was also not significant (Z = 1.53, *p* = 0.13). Between-study heterogeneity was very high (τ^2^ = 1.53; χ^2^ = 54.60, df = 4, *p* < 0.00001; I^2^ = 93%).

The hybrid/home-based subgroup included one study. The results showed no difference in changes in SRS-22 scores between the intervention and control groups (SMD = 0.00, 95% CI −0.72 to 0.72). The test for overall effect was not statistically significant (Z = 0.00, *p* = 1.00).

The test for subgroup differences indicated that the change in SRS-22 scores did not differ significantly between the two supervision groups (χ^2^ = 1.67, df = 1, *p* = 0.20). Accordingly, the current evidence does not support a differential effect of supervision intensity on SRS-22 outcomes.

### 3.6. Certainty of Evidence (GRADE)

The certainty of evidence for the main outcomes was assessed using the GRADE approach, and the domain-specific judgments and downgrade decisions are summarized in the main text and presented in detail in Supplementary Table S1.

The certainty of evidence for the Cobb angle was rated as moderate. The evidence was downgraded by one level because of risk-of-bias concerns in some included studies. Although moderate heterogeneity was observed (I^2^ = 58%), the direction and statistical significance of the effect were relatively consistent; therefore, no additional downgrading for inconsistency was applied.

The certainty of evidence for ATR was rated as low. Although a statistically significant effect was observed, the evidence was downgraded by one level for risk-of-bias concerns and by one level for inconsistency due to high heterogeneity (I^2^ = 86%).

The certainty of evidence for SRS-22 was rated as low. No statistically significant difference was observed, and the evidence was downgraded by one level for inconsistency due to very high heterogeneity (I^2^ = 91%) and by one level for imprecision related to the limited number of studies, relatively small sample size, and confidence intervals crossing no effect. The corresponding sensitivity analyses for the main outcomes are summarized in Supplementary Table S2.

## 4. Discussion

### 4.1. Principal Findings

This systematic review and meta-analysis comprehensively evaluated the effects of physiotherapeutic scoliosis-specific exercises (PSSE) in patients with adolescent idiopathic scoliosis (AIS), with particular emphasis on differences according to supervision intensity. Based on pooled data from 10 randomized controlled trials, PSSE significantly reduced the Cobb angle and angle of trunk rotation (ATR) compared with control interventions. In contrast, no statistically significant difference was observed in health-related quality of life as measured by the SRS-22 questionnaire.

In subgroup analyses according to supervision intensity, more consistent and larger effects were observed in fully supervised interventions. However, the statistical support for a subgroup effect differed across outcomes: the subgroup difference was statistically significant for ATR, whereas it did not reach statistical significance for Cobb angle and was not significant for SRS-22. Accordingly, the possible influence of supervision intensity should be interpreted as more strongly supported for ATR and as suggestive rather than definitive for Cobb angle.

This focus distinguishes the present review from earlier syntheses that primarily addressed the general effectiveness of PSSE. By restricting inclusion to randomized controlled trials and examining supervision intensity as a clinically meaningful subgroup factor, the present review provides a more methodologically focused synthesis and extends the literature beyond the general question of whether PSSE is effective. In this respect, the present review refines previous conclusions in a clinically relevant way [29,30]. In particular, it adds a perspective that is less developed in earlier quantitative reviews such as Ma et al. [29]. while remaining broadly compatible with the broader methodological overview provided by Seleviciene et al. [30].

### 4.2. Effects of PSSE on Structural Outcomes in AIS

The Cobb angle is the principal parameter for assessing structural progression in AIS [31]. The moderate effect size observed in this study suggests that PSSE may have beneficial effects on structural spinal alignment beyond mere functional improvement. Because the ultimate goal of AIS management is to prevent curve progression and minimize the need for surgery, these findings may have clinically relevant implications. However, they should be interpreted in light of the moderate certainty of evidence for Cobb angle [30]. These results are broadly consistent with more recent quantitative syntheses, particularly Ma et al. (2023), which also supported the structural benefits of PSSE in children and adolescents with idiopathic scoliosis [29]. By contrast, Seleviciene et al. (2022) provided a broader methodological overview of PSSE approaches and their clinical applications rather than a focused synthesis restricted to randomized controlled trials [30].

A significant reduction was also observed in ATR, with a more pronounced effect in the fully supervised intervention group. However, this finding should be interpreted more cautiously because the certainty of evidence for ATR was low and heterogeneity was substantial. As a clinical indicator reflecting trunk rotational deformity, ATR may be directly associated with the underlying mechanisms of PSSE, which include three-dimensional self-correction, rotational breathing, and trunk realignment training [9]. Although the ATR findings may suggest that closer supervision supports more effective structural correction, this interpretation remains cautious because of the substantial heterogeneity across studies and the exploratory nature of the subgroup analysis.

### 4.3. Effects on Health-Related Quality of Life

No statistically significant difference was observed in SRS-22 scores, and heterogeneity was very high. This finding suggests that quality-of-life measures are not necessarily directly associated with structural changes. Health-related quality of life in patients with AIS is influenced by multidimensional factors, including body image perception, psychological adaptation, peer relationships, and treatment satisfaction [32]. Moreover, most of the included studies were limited to short-term follow-up, and it is possible that structural improvements may not yet have translated into measurable psychosocial benefits.

Therefore, to more comprehensively evaluate the effects of PSSE from a patient-centered perspective, long-term follow-up studies and domain-specific analyses of patient-reported outcomes are warranted.

### 4.4. Supervision Intensity as a Potential Effect Modifier

One of the most important contributions of this study is the explicit evaluation of supervision intensity as a potential effect modifier. While previous reviews have mainly focused on whether PSSE is effective overall, the present review specifically examined how differences in intervention delivery, particularly the degree of therapist supervision, may influence treatment outcomes in randomized controlled trials.

In fully supervised settings, immediate feedback and individualized correction can be provided during each session. This may be particularly important in AIS, where the deformity is three-dimensional and precise postural adjustment is essential for effective structural correction. Thus, supervision intensity should not be interpreted simply as a difference in session frequency alone, but rather as a broader combination of direct therapist contact, real-time corrective feedback, adherence monitoring, and support for exercise fidelity. By contrast, hybrid/home-based interventions may allow less control over adherence and execution accuracy and may differ not only in exercise frequency or delivery mode, but also in the continuity of therapist oversight and the quality of neuromuscular re-education.

At the same time, the hybrid/home-based category should be interpreted cautiously because it included heterogeneous intervention formats, with varying combinations of therapist-supervised sessions, home exercise programs, and digitally delivered components. Therefore, the subgroup findings for this category should not be interpreted as representing a single uniform intervention model, and overgeneralization should be avoided.

Taken together, these subgroup findings should be interpreted as exploratory rather than confirmatory. In particular, the subgroup difference for Cobb angle did not reach statistical significance, and heterogeneity across studies remained present. Therefore, although supervision intensity may influence treatment effectiveness, the current evidence does not support a definitive conclusion regarding its modifying role. This caution is further warranted because subgroup analyses generally have limited statistical power and the present findings were derived from multiple exploratory comparisons across outcomes.

### 4.5. Methodological Considerations and Certainty of Evidence

The overall methodological quality of the included studies was generally acceptable; however, concerns were identified in some trials regarding deviations from intended interventions and selective reporting. In exercise-based intervention studies, blinding of participants and therapists is inherently challenging, and home-based programs may present additional limitations in monitoring adherence. These factors may contribute to an increased risk of bias.

According to the GRADE assessment, the certainty of evidence was rated as moderate for the Cobb angle, low for ATR, and low for SRS-22. These findings indicate that relatively consistent effects were observed for structural outcomes, whereas greater uncertainty remains for subjective outcomes. In particular, risk-of-bias concerns, high heterogeneity, and limited sample sizes contributed to downgrading across outcomes. For Cobb angle, the certainty was downgraded primarily because of risk-of-bias concerns in some included studies. For ATR, the certainty was downgraded because of both risk of bias and inconsistency related to substantial heterogeneity. For SRS-22, the certainty was downgraded because of inconsistency and imprecision, particularly due to very high heterogeneity, limited sample size, and confidence intervals crossing no effect.

The observed heterogeneity likely reflects both clinical and methodological differences across studies, including variations in concurrent brace use, baseline curve severity, intervention duration, and the type of PSSE delivered. Studies differed in whether PSSE was delivered as Schroth-based, SEAS-based, modified, or digitally supported programs, and some interventions were combined with bracing or other exercise approaches. These differences may have contributed to the substantial heterogeneity observed particularly in the ATR and SRS-22 analyses. Therefore, these pooled estimates should be interpreted with caution, especially for patient-reported outcomes, and their generalizability may be limited. In additional sensitivity analyses excluding studies judged as overall high risk of bias, the pooled effects for Cobb angle and ATR remained statistically significant and were essentially unchanged in magnitude and direction compared with the main analyses. For SRS-22, the pooled effect remained non-significant, although the direction of effect continued to favor the PSSE group and the magnitude of effect was slightly attenuated. These findings suggest that the overall structural benefits of PSSE for Cobb angle and ATR, as well as the non-significant result for SRS-22, were not driven solely by the inclusion of high-risk studies.

### 4.6. Clinical Implications

From a clinical perspective, these findings may support the use of PSSE as part of conservative management for adolescents with idiopathic scoliosis [13]. This interpretation is broadly consistent with previous reviews supporting PSSE as a conservative treatment option. At the same time, the present review adds a more clinically specific perspective by restricting the synthesis to randomized controlled trials and examining supervision intensity as a potential source of variability in treatment effects [29,30]. However, because the certainty of evidence was moderate for Cobb angle and low for ATR and SRS-22, and because substantial heterogeneity was observed in some analyses, these findings should be interpreted cautiously. Although more favorable effects were observed in fully supervised interventions, the evidence for a supervision-related difference was stronger for ATR than for Cobb angle and was not significant for SRS-22. Accordingly, any clinical recommendations based on the present review should be considered provisional until confirmed by larger, methodologically robust randomized controlled trials.

However, structural improvement does not necessarily translate directly into enhanced quality of life [13]. Therefore, when establishing treatment goals, an integrated approach that considers morphological, functional, and psychosocial objectives is warranted.

### 4.7. Limitations and Future Directions

This study has several limitations. The number of included studies was relatively small, and moderate to substantial heterogeneity was observed for some outcomes. In addition, clinical heterogeneity was present in terms of intervention duration, supervision structure, use of concomitant bracing, and participants’ stages of growth. Furthermore, most of the included studies were limited to short-term follow-up, which restricted the ability to fully evaluate the long-term effects of PSSE on curve progression prevention. Another methodological limitation is that data extraction and risk-of-bias assessment were not conducted independently by two reviewers. Instead, these steps were primarily performed by one reviewer, with consultation and verification by a co-investigator when clarification was needed. Although this process helped reduce potential errors, the absence of fully independent duplicate assessment may have introduced subjectivity and increased the risk of selection bias and information bias.

In addition, some meta-analyses initially included multiple curve-specific estimates derived from the same study. To address this issue, sensitivity analyses were conducted using one estimate per study for Cobb angle and ATR. The results remained statistically significant and directionally consistent with the main analyses; however, particularly for ATR, substantial heterogeneity persisted. Additional sensitivity analyses excluding studies judged as overall high risk of bias showed that the pooled effects for Cobb angle and ATR remained statistically significant and essentially unchanged, whereas the pooled effect for SRS-22 remained non-significant. These findings suggest that the main conclusions were not driven solely by the inclusion of high-risk studies, although heterogeneity remained substantial in some analyses.

The supervision-based subgroup findings should also be interpreted with caution. The hybrid/home-based supervision category remained heterogeneous, as it included different combinations of supervised sessions, home exercise programs, and digital interventions. This variability may have limited the interpretability of the subgroup findings. Moreover, the subgroup findings regarding supervision intensity should be considered exploratory, as the subgroup difference for Cobb angle was not statistically significant and heterogeneity remained across studies.

Future research should include large-scale, multicenter randomized controlled trials to determine the optimal supervision frequency and dose–response relationship of PSSE interventions. Comparative studies examining digitally delivered remote supervision models versus face-to-face supervision are also warranted, along with long-term follow-up investigations and cost-effectiveness analyses. Such approaches may help define the clinical applicability and practical effectiveness of PSSE more clearly.

## 5. Conclusions

This systematic review and meta-analysis suggest that physiotherapeutic scoliosis-specific exercises (PSSE) may improve structural spinal curvature and trunk rotational deformity in patients with adolescent idiopathic scoliosis (AIS). Reductions were observed in both the Cobb angle and ATR; however, the certainty of evidence was moderate for the Cobb angle and low for ATR.

Subgroup analyses according to supervision intensity showed larger and more consistent effects in fully supervised interventions. However, this pattern was more clearly supported for ATR than for Cobb angle, for which the subgroup difference did not reach statistical significance. Accordingly, the possible influence of supervision intensity should be regarded as suggestive rather than definitive.

No clear differential effect was observed for the quality-of-life outcome (SRS-22), for which the certainty of evidence was low, and substantial heterogeneity and methodological limitations remained in some analyses. Therefore, the clinical implications of the present findings should be considered provisional, particularly for outcomes beyond structural measures. Additional high-quality studies are needed to clarify the effects of PSSE on patient-centered outcomes.

Future research should determine the optimal supervision frequency and intervention dosage, evaluate the long-term sustainability of curve progression prevention through extended follow-up, and promote more standardized intervention protocols and reporting frameworks to strengthen the clinical applicability and evidence base of PSSE.

## Figures and Tables

**Table 1:** Characteristics of Included Studies and Intervention Protocols.

Study	Country	Intervention	Sample Size		Baseline Cobb Angle (°)	Outcome	min/day	Week	Session/Week	Supervision Intensity
		(I/C)	N (M/F)	Age						
Büyükturan et al. 2024 [21]	Turkey	Schroth	15 (3/12)	14.0 ± 1.9	T (22.8 ± 3.1) L (18.3 ± 3.7)	Cobb angle, ATR, SRS-22, WRVAS	90	24	3	Fully supervised
		Lyon	16 (4/12)	14.2 ± 2.0	T (20.4 ± 2.2) L (17.1 ± 1.9)					
Dimitrijevic et al. 2025 [22]	Serbia	supervised Schroth	17 (5/12)	14.11 ± 1.02	30.18 ± 8.19	Cobb angle, ATR, VC, FVC, FEV1, FEV1/FVC, CE	90	8	3	Fully supervised
		home Schroth	17 (5/12)	13.41 ± 1.63	30.24 ± 6.51		NR		NR	None
Kaya et al. 2025 [23]	Turkey	Schroth	34 (10/24)	13.8 ± 1.6	16.2 ± 2.7	Cobb angle, SRS-22, ATR, WRVAS	60	24	3	Fully supervised
		PNF	33 (8/25)	14.1 ± 1.8	17.2 ± 4.2					
Khaledi et al. 2024 [24]	Iran	Schroth	15 (15/0)	16.27 ± 1.44	15.09 ± 4.41	Cobb angle, ATR, SRS-22	50–70	12	3	Fully supervised
		Schroth + SS	15 (15/0)	16.33 ± 0.9	16.45 ± 5.25				3	Fully supervised
		control	10 (10/0)	15.4 ± 1.51	11.32 ± 0.96				None	None
Kocaman et al. 2021 [12]	Turkey	Schroth + TE	14 (4/10)	14.07 ± 2.37	T (17.64 ± 4.01) L (15.80 ± 3.42)	Cobb angle, WRVAS, ATR, SRS-22, SM, PMS	90	10	3	Fully supervised
		Core + TE	14 (3/11)	14.21 ± 2.19	T (17.29 ± 3.45) L (15.17 ± 4.02)					
Kyrkousis et al. 2024 [25]	Greece	Schroth + brace	40 (4/36)	13.67 ± 1.19	33.22 ± 6.27	Cobb angle, Sum of Curves, ATR, SRS-22	60	12	3	Fully supervised
		brace	40 (4/36)	13.55 ± 1.02	34.62 ± 3.14				None	None
Mohamed et al. 2021 [26]	Egypt	Schroth	17 (0/17)	14.5 ± 1.2	20.42 ± 2.57	Cobb angle, ATR, TSPP, 6MWT	60	24	3	Fully supervised
		PNF	17 (0/17)	14.9 ± 1.4	20.21 ± 2.8					
Schreiber et al. 2016 [13]	Canada	Schroth + SC	25 (2/23)	13.4 ± 1.6	27.9 ± 8.8	Cobb angle, ATR, SRS-22r	30–45	24	1 supervised + daily home	Hybrid/home-based
		SC	25 (1/24)	13.7 ± 1.5	29.1 ± 8.9		None		None	None
Yagci and Yakut 2019 [27]	Turkey	SEAS + brace	15(0/15)	14.2 ± 1.5	T (27.6 ± 8) L (25.9 ± 8)	Cobb angle, ATR, WRVAS, POSTI, SRS-22	40 + 20 (home)	16	1 supervised + daily home	Weekly supervised
		Core + brace	15 (0/15)	14 ± 1.3	T (30 ± 9.3) L (24.9 ± 9)					
Yuan et al. 2025 [28]	China	Digital PSSE	64 (15/49)	10.95 ± 2.13	15.78 ± 3.38	Cobb angle, ATR, POA	30	24	≥5 home + 1 initial supervised	Digital + therapist
		Conventional PSSE	64 (16/48)	11.31 ± 2.33	16.03 ± 3.45		NR			Limited in-person

M: Male, F: Female, I: Intervention, C: Control, T: Thoracic, L: Lumbar, 6MWT: Six-minute walk test, ATR: angle of trunk rotation, CE: chest expansion, FEV1: forced expiratory volume in the first second, FVC: forced vital capacity, NR: Not Reported, PMS: Peripheral Muscle Strength, PNF: Proprioceptive Neuromuscular Facilitation, POA: Pelvic obliquity angle, POSTI: Posterior Trunk Symmetry Index, SC: Standard of Care, SM: Spinal mobility, SS: Spinal Stabilization exercise, SRS-22: Scoliosis Research Society-22, TE: Traditional exercises, VC: vital capacity, WRVAS: Walter Reed Visual Assessment Scale.

## Data Availability

All data analyzed in this study are included in the published article and its Supplementary Materials.

## Supplementary Materials

The following supporting information can be downloaded at: https://www.mdpi.com/article/10.3390/medicina62040768/s1, Table S1: Summary of Findings and GRADE Assessment for Main Outcomes. Table S2: Results of sensitivity analyses for Cobb angle, ATR, and SRS-22, including one-estimate-per-study analyses and analyses excluding studies judged as overall high risk of bias.

## Abbreviations

M: Male, F: Female, I: Intervention, C: Control, 6MWT: Six-minute walk test, ATR: angle of trunk rotation, CE: chest expansion, FEV1: forced expiratory volume in the first second, FVC: forced vital capacity, NR: Not Reported, PMS: Peripheral Muscle Strength, PNF: Proprioceptive Neuromuscular Facilitation, POA: Pelvic obliquity angle, POSTI: Posterior Trunk Symmetry Index, SM: Spinal mobility, SRS-22: Scoliosis Research Society-22, VC: vital capacity, WRVAS: Walter Reed Visual Assessment Scale.
