# Supplementary material for: Effect of Supervised Versus Hybrid Delivery of Physiotherapeutic Scoliosis-Specific Exercises in Adolescents with Idiopathic Scoliosis: Systematic Review and Meta-Analysis
Source: Medicina (Kaunas). 2026 Apr 15;62(4):768. doi: 10.3390/medicina62040768 (PMC13117688; doi:10.3390/medicina62040768)
Supplement: Supplementary file 1 [file medicina-62-00768-s001.zip › medicina-4208043-supplementary.pdf]

Supplementary Table S1. Summary of Findings and GRADE Assessment for Main Outcomes.

Outcome	No. of Studies	Effect Estimate	Risk of Bias	Inconsistency	Indirectness	Imprecision	Publication Bias	Overall Certainty	Reason for Downgrade
**Cobb angle**	10 studies	SMD −0.52, 95% CI −0.79 to −0.25	Serious	Not serious	Not serious	Not serious	Undetected	Moderate	Downgraded by 1 level for risk of bias in some included studies.
**ATR**	9 studies	SMD −1.01, 95% CI −1.53 to −0.48	Serious	Serious	Not serious	Not serious	Undetected	Low	Downgraded by 1 level for risk of bias and 1 level for inconsistency due to substantial heterogeneity.
**SRS-22**	6 studies	SMD 0.73, 95% CI −0.22 to 1.68	Not serious	Serious	Not serious	Serious	Undetected	Low	Downgraded by 1 level for inconsistency due to very high heterogeneity and 1 level for imprecision due to limited sample size and wide confidence interval.

Abbreviations: ATR, angle of trunk rotation; CI, confidence interval; GRADE, Grading of Recommendations Assessment, Development and Evaluation; SMD, standardized mean difference; SRS-22, Scoliosis Research Society-22.

Evidence from randomized controlled trials was initially rated as high certainty and downgraded based on the GRADE domains of risk of bias, inconsistency, indirectness, imprecision, and publication bias. Publication bias was not formally assessed because of the limited number of included studies per outcome.

Supplementary Table S2. Results of sensitivity analyses for Cobb angle, ATR, and SRS-22, including one-estimate-per-study analyses and analyses excluding studies judged as overall high risk of bias.

Outcome	Main Analysis	One-Estimate-per-Study Sensitivity Analysis	Excluding High-Risk Studies Sensitivity Analysis	Reason for Downgrade
**Cobb angle**	SMD = −0.52 (95% CI −0.79 to −0.25), I^2 = 58%	SMD = −0.63 (95% CI −0.90 to −0.36), I^2 = 48%	SMD = −0.51 (95% CI −0.83 to −0.19), I^2 = 63%	The pooled effect remained statistically significant and directionally consistent across analyses, suggesting that the overall effect on Cobb angle was robust to both the handling of multiple estimates and the exclusion of high-risk studies.
**ATR**	SMD = −1.01 (95% CI −1.53 to −0.48), I^2 = 86%	SMD = −1.20 (95% CI −1.81 to −0.59), I^2 = 88%	SMD = −0.99 (95% CI −1.59 to −0.38), I^2 = 86%	The pooled effect remained statistically significant and directionally consistent across analyses. However, considerable heterogeneity persisted, indicating that the ATR findings were robust but should still be interpreted cautiously.
**SRS-22**	SMD = 0.73 (95% CI −0.22 to 1.68), I^2 = 91%	Not applicable	SMD = 0.58 (95% CI −0.50 to 1.66), I^2 = 90%	The pooled effect remained non-significant, although the direction of effect continued to favor the PSSE group. This suggests that the non-significant result for SRS-22 was not materially altered by the exclusion of high-risk studies.
